# Proteomic Identification of Potential Target Proteins of Cathepsin W for Its Development as a Drug Target for Influenza

**DOI:** 10.1128/spectrum.00921-22

**Published:** 2022-07-14

**Authors:** Sira C. Günther, Carles Martínez-Romero, Milagros Sempere Borau, Christine T. N. Pham, Adolfo García-Sastre, Silke Stertz

**Affiliations:** a Institute of Medical Virology, University of Zurich, Zurich, Switzerland; b Life Science Zurich Graduate School, ETH and University of Zurich, Zurich, Switzerland; c Department of Microbiology, Icahn School of Medicine at Mount Sinai, New York, New York, USA; d Global Health and Emerging Pathogens Institute, Icahn School of Medicine at Mount Sinai, New York, New York, USA; e Department of Medicine, Division of Rheumatology, Washington University School of Medicine, St. Louis, Missouri, USA; f Department of Medicine, Icahn School of Medicine at Mount Sinai, New York, New York, USA; g The Tisch Cancer Institute, Icahn School of Medicine at Mount Sinai, New York, New York, USA; h Department of Pathology, Molecular and Cell-Based Medicine, Icahn School of Medicine at Mount Sinai, New York, New York, USA; Erasmus MC

**Keywords:** cysteine protease cathepsin W, CTSW, influenza A virus, terminal amine isotopic labeling of substrates, TAILS, epsin 2, EPN2, host-directed antivirals, cathepsin W, influenza virus, virus entry

## Abstract

Influenza A virus (IAV) coopts numerous host factors for efficient replication. The cysteine protease cathepsin W (CTSW) has been identified as one host factor required for IAV entry, specifically for the escape of IAVs from late endosomes. However, the substrate specificity of CTSW and the proviral mechanism are thus far unknown. Here, we show that intracellular but not secreted CTSW promotes viral entry. We reveal 79 potential direct and 31 potential indirect cellular target proteins of CTSW using the high-throughput proteomic approach terminal amine isotopic labeling of substrates (TAILS) and determine the cleavage motif shared by the substrates of CTSW. Subsequent integration with data from RNA interference (RNAi) screens for IAV host factors uncovers first insights into the proviral function of CTSW. Notably, CTSW-deficient mice display a 25% increase in survival and a delay in mortality compared to wild-type mice upon IAV infection. Altogether, these findings support the development of drugs targeting CTSW as novel host-directed antiviral therapies.

**IMPORTANCE** Influenza viruses are respiratory pathogens and pose a constant threat to human health. Although antiviral drugs are available for influenza, the emergence and spread of drug-resistant viruses is cause for concern. Therefore, the development of new antivirals with lower chances of their target viruses acquiring resistance is urgently needed to reduce the high morbidity and mortality caused by influenza. Promising alternatives to drugs targeting viral proteins are those directed against host factors required for viral replication. The cysteine protease cathepsin W (CTSW) is an important host factor for IAV replication, and its proteolytic activity is required for fusion of viral and endosomal membranes. In this work, we identify a number of hitherto unknown CTSW substrates, providing new insights into virus-host interactions, and reveal that CTSW might also play a proviral role in an *in vivo* model. These results support the development of CTSW as a drug target for next-generation antivirals against influenza.

## INTRODUCTION

Influenza is a highly infectious respiratory disease that spreads globally, mainly in the winter season, causing 3 to 5 million severe infections and up to 650,000 deaths worldwide each year (1). Current seasonal influenza virus vaccines and antiviral drugs are available but only partially effective due to rapid changes in the viral epitopes targeted by the vaccine and the emergence and spread of drug-resistant viruses (2). To date, there are three classes of drugs to treat influenza A virus (IAV) infections: the neuraminidase inhibitors (NAIs), the M2 ion channel inhibitors, and the relatively new polymerase acidic (PA) endonuclease inhibitor. Currently, widespread resistance to adamantanes (M2 inhibitors) is present in circulating viruses and resistance to all NAIs is a concern (3). Therefore, the development of new antivirals with lower chances of acquiring resistance is urgently needed. Given that viruses hijack host cell proteins to replicate, a thorough understanding of this intricate virus-host interlay is of paramount importance to develop novel anti-influenza drugs targeting host factors involved in virus replication (4).

In this study, we analyzed the role of the host cell protease cathepsin W (CTSW) in IAV entry, as we had previously found that knockdown (KD) of CTSW expression led to entrapment of IAV in the late endosome due to a block at the stage of viral fusion (5). Moreover, we had shown that the proteolytic activity of CTSW is required for the escape of IAVs from late endosomes, as overexpression of CTSW wild type (CTSW WT) but not the enzymatically inactive mutant (CTSW mut) could rescue the effect of small interfering RNA (siRNA)-mediated knockdown of CTSW (5). CTSW belongs to a family of cysteine proteases, comprising 11 members in humans (6). Cysteine cathepsins are lysosomal proteases characterized by the presence of a cysteine in their catalytic triad. To prevent any uncontrolled proteolytic activity, cysteine cathepsins are synthesized as inactive preproenzymes with a signal peptide (SP) targeting them to the endoplasmic reticulum (ER) and an inhibitory propeptide directing them to the lysosome and enabling proper folding. For activation of the mature enzyme, the propeptide that acts as an endogenous inhibitor needs to be cleaved off autocatalytically or by another enzyme (7, 8). For a long time, nonspecific protein degradation in the endolysosomal system was considered the only function of cysteine cathepsins. However, this view is changing, as there is now clear evidence for their localization in other cellular compartments and, thus, involvement in several physiological but also pathological processes. Moreover, due to lysosomal exocytosis, secreted cysteine cathepsins are emerging as important players in the extracellular space (9, 10).

Given the requirement of the proteolytic activity of CTSW for successful IAV entry, we considered CTSW a promising target for the development of host-directed antivirals. To further develop CTSW as a potential drug target, knowledge about the proteolytic targets of CTSW and the proviral mechanism of action would be useful. Here, we employed a high-throughput proteomic approach called terminal amine isotopic labeling of substrates (TAILS) to identify proteolytic targets of CTSW and used integration with data from RNA interference (RNAi) screens to gain first insights into the proviral mechanism of action. Moreover, we show that CTSW knockout (KO) mice display reduced mortality and pathogenicity upon IAV infection and, thus, provide *in vivo* evidence for the suitability of CTSW as a novel influenza drug target.

## RESULTS

### Intracellular rather than secreted CTSW exerts its proviral function in IAV entry.

**FIG 1:**
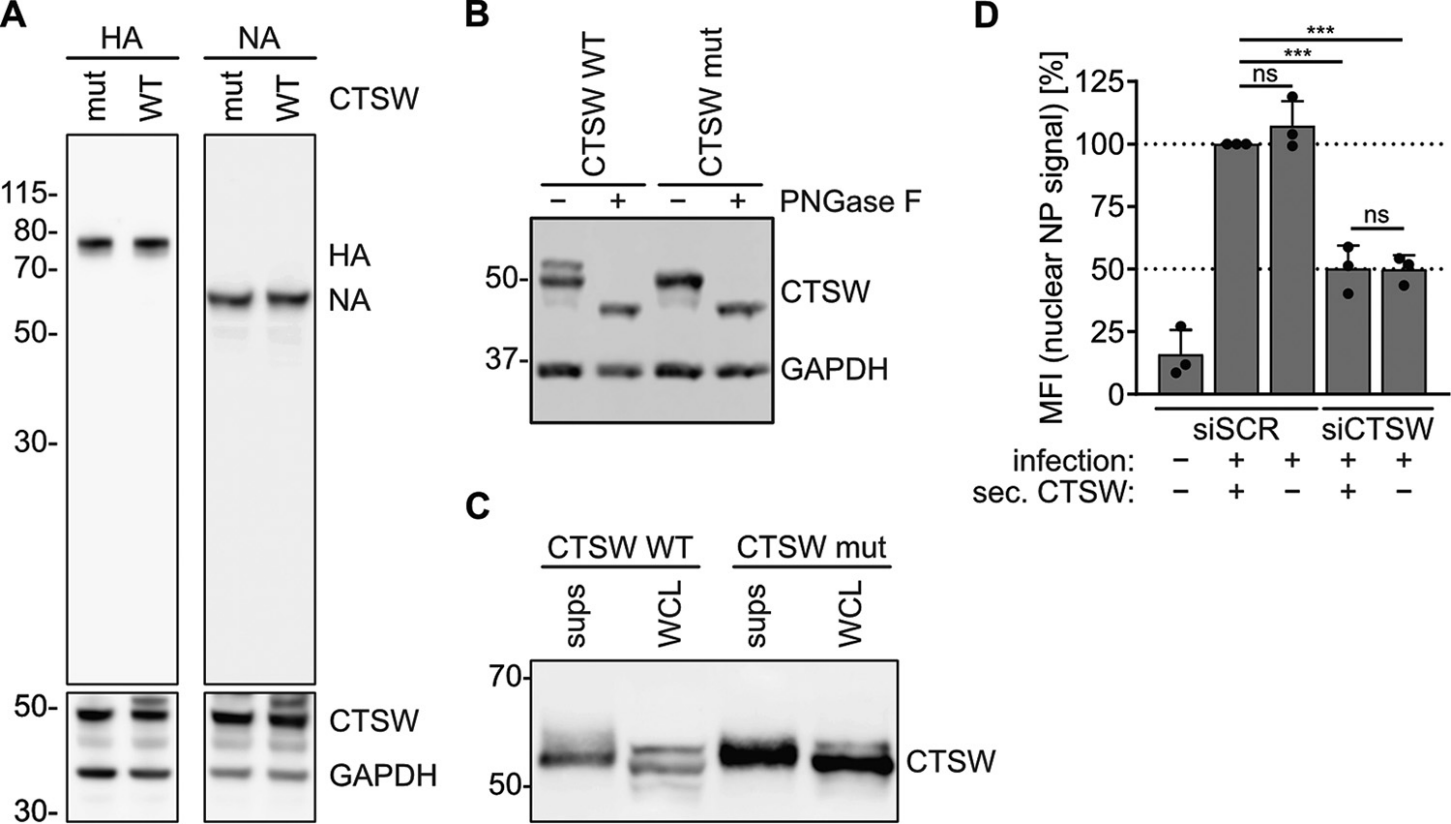
Intracellular rather than secreted CTSW exerts its proviral function in IAV entry. (A) HEK293T cells cotransfected with plasmids encoding viral proteins (HA and NA) and wild-type CTSW (CTSW WT) or an enzymatically inactive mutant of CTSW (CTSW mut) were lysed, and the indicated proteins were detected by Western blot analysis using monoclonal mouse anti-HA, polyclonal rabbit anti-NA, polyclonal rabbit anti-GAPDH, and monoclonal mouse anti-CTSW antibodies. (B) HEK293T cells were transfected with the indicated CTSW expression constructs. At 24 h posttransfection, cells were lysed in radioimmunoprecipitation assay (RIPA) buffer, left untreated (−) or treated with PNGase F (+) to remove *N*-linked oligosaccharides from glycoproteins, and subjected to Western blot analysis to detect CTSW and GAPDH as described in the legend to panel A. (C) HEK293T cells were transfected with plasmids encoding either His-tagged CTSW WT or mut. His-CTSW WT and mut were purified from the cell extracts (whole-cell lysates [WCL]) or the supernatants (sups) of the transfected cells via nickel Sepharose beads and subjected to Western blot analysis for CTSW using a monoclonal mouse anti-CTSW antibody. (D) A549 cells were transfected with a nontargeting (scrambled) siRNA (siSCR) or an siRNA targeting CTSW (siCTSW). At 24 h posttransfection, the transfection medium was changed to DMEM only, and another 24 h later, supernatant containing (siSCR) or lacking (siCTSW) secreted CTSW was harvested and preincubated with A/WSN/1933 (MOI = 10 PFU per cell) for 2 h at 37°C. siRNA-treated cells were then infected with preincubated virus as indicated. At 3 h postinfection, cells were fixed, stained for the viral nucleoprotein (NP) and nuclei, and analyzed by fluorescence microscopy. Mean fluorescence intensity (MFI) in the nuclei (nuclear NP signal) was quantified using ImageJ (*n* > 600) and normalized to that of cells treated with siSCR and infected with virus preincubated with secreted CTSW. Mean values from three independent experiments are plotted, error bars represent standard deviations (SDs), and individual data points are shown. Statistical testing was done using the unpaired *t* test: ***, *P* < 0.001; ns, not significant.

The proteolytic activity of human CTSW has been shown to be required for efficient IAV fusion in the late endosome, rendering CTSW a suitable drug target for host-directed antivirals (5). However, the proteolytic targets of CTSW are thus far unknown. CTSW must cleave or (partially) degrade either a viral protein or a cellular protein, facilitating the fusion of IAV with the endosomal membrane. While our previous study had shown that no cleavage of viral proteins could be detected for incoming virus particles, the possibility remained that hemagglutinin (HA) or neuraminidase (NA) could be cleaved by CTSW due to the low sensitivity of the approach used (5). To further test whether CTSW could cleave HA or NA, we cotransfected plasmids encoding HA or NA with CTSW wild type (CTSW WT) or an enzymatically inactive mutant of CTSW (CTSW mut) in which the cysteine at position 153 in the catalytic triad has been replaced by an alanine (Fig. 1A). Given that endosomal and ER localization have been described for CTSW and both HA and NA are trafficked through the ER, this assay could reveal potential cleavage of the viral glycoproteins. However, no cleavage product or reduction of protein levels could be detected for HA or NA. Of note, two to three bands were detected for CTSW WT and only one to two bands for CTSW mut on Western blots. Enzymatic deglycosylation revealed that the higher bands were glycosylated versions of CTSW (Fig. 1B).

In a next step, we tested whether CTSW could be secreted from the cell, as cleavage of viral proteins, in particular HA, could also occur in the extracellular space. When we expressed a tagged version of CTSW and purified it from cell extracts and supernatants, we detected CTSW bands in both cell extract and supernatant fractions, confirming that CTSW indeed got secreted (Fig. 1C). As there are no CTSW targets known so far, we could not test whether the secreted CTSW was proteolytically active, but we still evaluated the possibility that secreted CTSW interacted with the virion before it entered the host cell, rather than cellular CTSW. To address this, we incubated IAV with supernatant of cells expressing CTSW (CTSW-secreting cells treated with a nontargeting [scrambled] siRNA [siSCR] [siSCR, + sec. CTSW]) or of cells that were treated with an siRNA targeting CTSW (siCTSW, − sec. CTSW) prior to infection of control (siSCR) or CTSW knockdown (siCTSW) cells (Fig. 1D). Cells lacking CTSW (siCTSW, − sec. CTSW) showed a 50% reduction in the nuclear nucleoprotein (NP) signal compared to the nuclear NP signal in cells expressing CTSW (siSCR), as the virus is trapped in late endosomes in the absence of CTSW. However, infecting CTSW knockdown cells with virus preincubated with supernatant containing secreted CTSW (siCTSW, + sec. CTSW) did not rescue this phenotype. Thus, we conclude that the proviral function of CTSW is mediated by intracellular rather than extracellular CTSW and that CTSW exerts its function by cleaving a cellular rather than a viral protein.

### Proteomic approach identifies potential cellular target proteins of CTSW in IAV entry.

**FIG 2:**
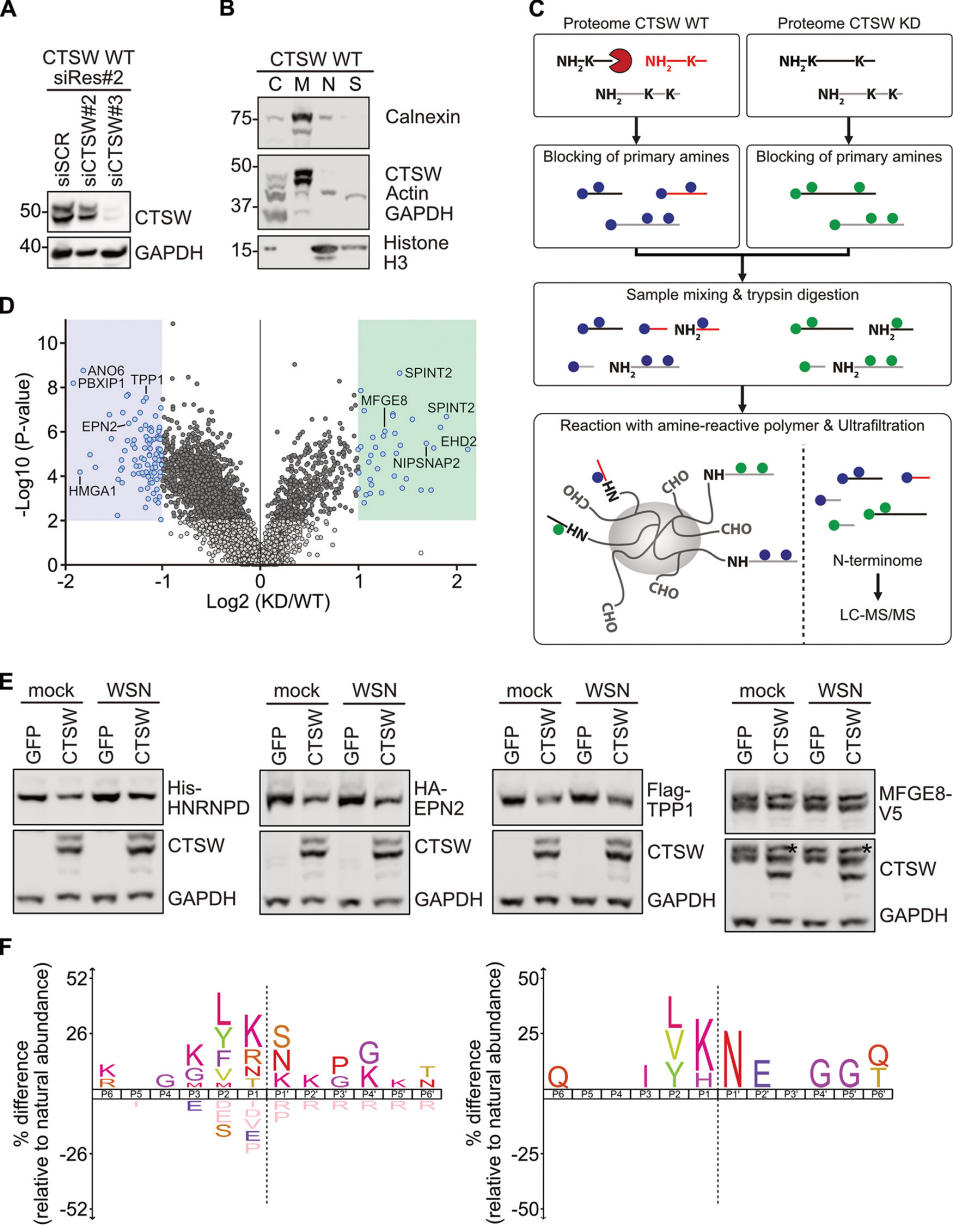
Identification of potential substrates of CTSW by TAILS. (A) Western blot analysis to assess siRNA knockdown efficiency in A549 cells stably overexpressing CTSW wild type, which is resistant to siRNA-mediated knockdown by siCTSW#2 (CTSW WT siRes#2). A nontargeting siRNA (siSCR) served as the negative control. (B) Subcellular fractionation of A549 cells stably overexpressing CTSW (CTSW WT) using the Qproteome cell compartment kit (Qiagen). The cytosolic fraction (F1; C, cytosol) primarily contains cytosolic proteins, the membrane fraction (F2; M, membrane) membrane proteins, the nuclear fraction (F3; N, nucleus) nuclear proteins, and the cytoskeletal fraction (F4; S, cytoskeleton) cytoskeletal proteins. GAPDH served as the marker for F1, calnexin for F2, histone H3 for F3, and actin for F4. (A, B) The indicated proteins were detected using monoclonal mouse anti-CTSW, monoclonal mouse anti-beta-actin, polyclonal rabbit anti-GAPDH, polyclonal rabbit anti-calnexin, and rabbit polyclonal anti-histone H3 antibodies. (C) TAILS (terminal amine isotopic labeling of substrates) workflow to compare the proteomes—more specifically, the proteomes of the membrane fraction—of CTSW WT and CTSW knockdown (KD) cells infected for 90 min with A/WSN/1933 (MOI = 10 PFU per cell). First, the natural and CTSW-generated N-terminal (NH_2_) and lysine (K) residues are blocked by demethylation, represented by blue (CTSW WT) or green (CTSW KD) dots. The two samples are then mixed and digested with trypsin, which generates internal tryptic peptides with free N termini. These free N termini react with an amine-reactive polymer, whereas the natural and CTSW-generated N termini, which are already blocked, will remain unbound and can be separated from the newly formed internal tryptic peptides by ultrafiltration. The enriched N-terminal peptides, also called the N terminome, are then analyzed and quantified by liquid chromatography-tandem mass spectrometry (LC-MS/MS). (D) Volcano plot displaying significantly altered N-terminal peptides in IAV-infected CTSW KD versus CTSW WT cells (log_2_ fold change [FC] of ≤−1 or ≥1, *P* < 0.01). Peptides that are less abundant in CTSW KD than in CTSW WT cells (direct targets) are shown on the left (blue dots in blue shaded area), and peptides that are more abundant in CTSW KD than in CTSW WT cells (indirect targets) are shown on the right (blue dots in green shaded area). Dark gray dots are significant and do not meet the FC threshold, light gray dots are nonsignificant and do not meet the FC threshold, and white dots are nonsignificant and greater than the upper FC threshold. (E) HEK293T cells cotransfected with constructs encoding tagged target proteins His-HNRNPD, HA-EPN2, Flag-TPP1, and MFGE8-V5 and CTSW or GFP as the negative control were infected for 90 min with A/WSN/1933 (MOI = 10 PFU per cell) or mock treated, and cell lysates were analyzed by Western blotting with tag-specific antibodies, a polyclonal rabbit anti-GAPDH antibody, and a monoclonal mouse anti-CTSW antibody. An asterisk indicates residual MFGE8 staining, as the blot was first stained against the V5 tag and then against CTSW and GAPDH. (F) Cleavage site specificity profiling of CTSW based on TAILS data of all 92 N-terminal peptides that were less abundant in CTSW KD than in CTSW WT cells (left) or of the 19 top-ranked N-terminal peptides (right). The iceLogo plots visualize the amino acid frequencies at P6 to P6′ positions of neo-N termini generated by CTSW with the human Swiss-Prot proteome as the reference set. Dotted lines indicate the cleavage site. Significantly overrepresented amino acids are shown above the *x* axis, underrepresented residues are shown below the *x* axis, and amino acids that have not been identified are depicted in salmon pink below the *x* axis (*P* = 0.05).

In order to identify cellular or viral proteins that are cleaved by CTSW and to study its cleavage pattern, we employed terminal amine isotopic labeling of substrates (TAILS), a high-throughput quantitative proteomic approach to discover protease substrates (11). To this end, we compared the N terminome of IAV-infected cells expressing endogenous CTSW (CTSW WT) and CTSW knockdown (CTSW KD) cells. As endogenous levels of CTSW are not detectable by Western blot analysis in A549 cells, we used A549 cells stably overexpressing CTSW that are resistant to one CTSW-specific siRNA (siCTSW#2; CTSW WT siRes#2) to validate knockdown efficiency (Fig. 2A). To enrich for proteins that are more likely to be cleaved by CTSW, we separated the cell lysates into subcellular fractions and only used fraction 2 (F2), containing membrane proteins. To confirm that the vast majority of CTSW was also present in F2, we again used CTSW-overexpressing cells (Fig. 2B). For the TAILS experiment, A549 cells transfected with a nontargeting siRNA (CTSW WT) or an siRNA targeting CTSW (CTSW KD) were infected with IAV (A/WSN/1933) for 90 min, as our previous work had revealed a pronounced effect of CTSW KD at this time point postinfection (5). The proteomes of CTSW WT and CTSW KD cells were then enriched for all N-terminal peptides, called the N terminome, and subjected to tandem mass spectrometry (MS/MS) (Fig. 2C). Analysis and quantification of CTSW-induced changes in the N terminome early in infection revealed 92 peptides that were less abundant in CTSW KD cells than in CTSW WT cells, and these represented potential direct targets (Fig. 2D, blue box, and Table S1 in the supplemental material). We also detected 37 peptides that were more abundant in CTSW KD cells than in CTSW WT cells, which represented potential indirect targets (Fig. 2D, green box, and Table S1). Of note, no peptides derived from viral proteins were among the hits. Assuming that indirect substrates would be more difficult to verify due to an unknown intermediate protease that was only proteolytically active in the absence of CTSW, we focused our attention on validating direct CTSW targets. Based on the availability of expression constructs and suitable antibodies, we tested several potential hits for cleavage by CTSW. Heterogeneous nuclear ribonucleoprotein D (HNRNPD), epsin 2 (EPN2), and tripeptidyl peptidase 1 (TPP1), among others, showed reduced protein levels when cotransfected with CTSW compared to their levels in the green fluorescent protein (GFP) control, regardless of infection (Fig. 2E and Fig. S1). To test whether the observed reductions in protein levels of direct CTSW substrates were specific, we also included one indirect CTSW target protein, milk fat globule epidermal growth factor 8 (MFGE8) (Fig. 2E and Fig. S1). As expected, the protein levels of MFGE8 were not reduced in the presence of CTSW. While overexpression of proteins comes with limitations, such as the possibility of altered localization, the results demonstrate that the reduction in protein levels is not unspecific.

Using the neo-N-terminal peptides generated by CTSW, we determined the preferred cleavage site motif of CTSW (Fig. 2F). The cleavage site of a protease is defined as …P3-P2-P1-P1′-P2′-P3′…, and cleavage occurs between the P1 and P1′ residues (12). Taking into account all 92 peptides referring to direct CTSW target proteins, we observed some preferences N and C terminal to the scissile bond (left motif). The high frequencies of leucine (L) and valine (V) at the P2 position and of arginine (R) at P1 are in line with a previous theoretical model predicting substrate specificities of CTSW (13). Strikingly, by only using the 19 top-ranked peptides out of a total of 92 peptides, there is a clear preference for arginine (N) at the P1′ and lysine (K) at the P1 position (right motif). Overall, these data reveal a large set of CTSW-specific substrates and provide first insights into the substrate specificity of CTSW.

### Integration of TAILS results with data from RNAi screens for IAV host factors.

**FIG 3:**
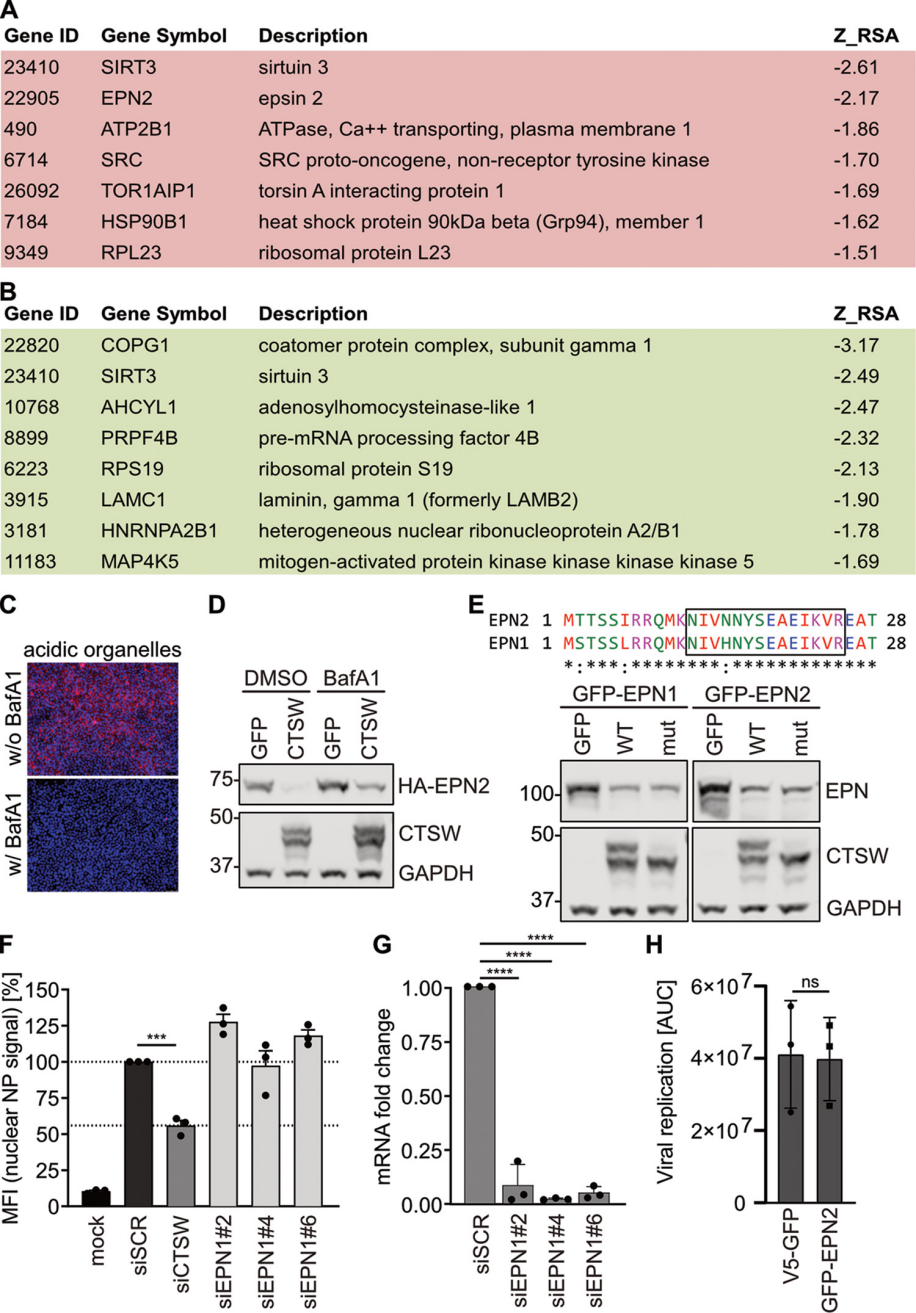
Integration of TAILS results with data from RNAi screens for IAV host factors. (A, B) List of potential direct targets of CTSW for which an antiviral (A) or a proviral (B) role in IAV infection was predicted by Metascape (15). (C) Representative images of HEK293T cells incubated with LysoTracker to label acidic organelles (red) in live cells treated with (w/) or without (w/o) 10 nM bafilomycin A1 (BafA1) for 16 h. DAPI was used to counterstain the nuclei (blue). (D) HEK293T cells cotransfected with HA-EPN2- and CTSW- or GFP-encoding plasmids were treated with DMSO or 40 nM bafilomycin A1 (BafA1) 4 h posttransfection for 20 h. Cell lysates were analyzed by Western blotting using polyclonal rabbit anti-GAPDH, monoclonal mouse anti-EPN2, and monoclonal mouse anti-CTSW antibodies. (E) Sequence alignment of epsin 2 (EPN2) and epsin 1 (EPN1) is shown at the top. The unique EPN2 peptide identified by TAILS (terminal amine isotopic labeling of substrates) and the corresponding peptide for EPN1 are boxed. Western blots analyzing HEK293T cells cotransfected with GFP-EPN1- or GFP-EPN2-expressing plasmids and constructs encoding GFP, CTSW WT, or CTSW mut are shown below. In addition to a monoclonal mouse anti-EPN1 antibody, the same antibodies as described in the legend to panel D were used. (F) A549 cells were transfected with a nontargeting siRNA (siSCR) or siRNAs targeting CTSW (siCTSW) or epsin 1 (siEPN1#2, #4, and #6). At 48 h posttransfection, cells were infected with A/WSN/1933 (MOI = 10 PFU per cell) for 1 h at 4°C, followed by 3 h at 37°C. Fixed and permeabilized cells were then stained for the viral nucleoprotein (NP) and nuclei and analyzed by fluorescence microscopy. Mean fluorescence intensity (MFI) in the nuclei (nuclear NP signal) was quantified using ImageJ (*n* > 400) and normalized to that of cells treated with siSCR. Mean values from three independent experiments are plotted, error bars represent standard errors of the means (SEMs), and individual data points are shown. (G) Knockdown efficiencies of three siRNAs targeting EPN1 were assessed by reverse transcription-qPCR analysis. GAPDH transcript levels were used for normalization. Shown are mean values from three independent experiments, each performed at least in duplicates. (H) A549-GFP (black symbols) and A549-GFP-EPN2 (pink symbols) cells were infected with WSN-Ren (MOI = 2), and *Renilla* luciferase expression was monitored with the live-cell substrate EnduRen at the indicated time points. The viral growth curves were measured in three independent experiments, and the area under the curve (AUC) was determined. (F to H) Statistical testing was done using the unpaired *t* test: ***, *P* < 0.001; ****, *P* < 0.0001; ns, nonsignificant.

In order to choose potential target proteins of CTSW for follow-up studies on IAV entry, we used Metascape, a portal based on meta-analysis of influenza OMICs data sets, to select for those factors that are predicted to affect IAV infection efficiency (14, 15). We again focused on direct targets of CTSW and hypothesized that one or more of them would have restrictive potential for IAV. Being degraded by CTSW would reduce their restrictive effect and, thus, allow viral entry to proceed. All potential direct targets were therefore analyzed for their restriction scores via Metascape, and targets with a score of less than −1.5 were considered hits. This resulted in seven direct CTSW targets with restrictive potential (Fig. 3A). Alternatively, a direct target of CTSW could require cleavage for activation of its proviral role, leading us to also analyze the direct targets for a potential proviral function using Metascape. This revealed eight genes with a predicted positive role in IAV infection that could potentially be linked to CTSW function (Fig. 3B). From the 7 restrictive and the 8 proviral factors, epsin 2 (EPN2) stood out as the one with a described function in endocytosis, and thus, EPN2 was selected for follow-up experiments.

As our TAILS validation experiments had revealed reduced EPN2 levels upon CTSW expression (Fig. 2E), we hypothesized that CTSW led to lysosomal degradation of EPN2. We tested whether treatment with bafilomycin A1 (BafA1), a specific inhibitor of vacuolar-type H^+^-ATPases (V-ATPases) that inhibits acidification and protein degradation in organelles (16), could restore EPN2 levels in the presence of CTSW. We verified that BafA1 led to a strong reduction in acidic organelles, as indicated by LysoTracker staining (Fig. 3C). When testing EPN2 levels in the presence of CTSW, we found that, indeed, BafA1 treatment resulted in increased EPN2 levels (Fig. 3D). This effect was most pronounced in CTSW-overexpressing cells but was also observed in control cells, possibly due to endogenous CTSW levels. Given that epsins are a conserved family and the EPN2 cleavage site is located in the highly conserved epsin N-terminal homology (ENTH) domain (Fig. 3E), we investigated whether the specificity of CTSW also includes epsin 1 (EPN1) and whether tagging epsins with GFP at the N terminus would result in a bigger cleavage product that could be detected. Cotransfection of EPN1 and CTSW led to reduced EPN1 protein levels compared to cotransfection of the GFP control, but no cleavage product could be detected (Fig. 3E). However, coexpression of the catalytically inactive mutant of CTSW had the same effect as coexpression of CTSW WT, both for EPN1 and for EPN2 (Fig. 3E). These data indicate that EPN2 and EPN1 can be degraded in a CTSW-dependent manner but that this might be independent of the proteolytic activity of CTSW.

Next, we aimed to reveal whether either EPN1 or EPN2 was functionally linked to the proviral role of CTSW. Based on the Metascape results, we expected EPN2 to show a restrictive effect, but for EPN1, we anticipated a proviral function, as EPN1 has been shown to be required for clathrin-dependent endocytosis of IAV (17). In a first step, we assessed whether siRNA-mediated knockdown of EPN1 reduced IAV entry efficiency, but in line with the described redundancy in entry routes for IAV (17, 18), we did not observe any inhibition of IAV entry upon EPN1 downregulation (Fig. 3F), despite efficient knockdown (Fig. 3G). In a next step, we generated stable A549 cell lines overexpressing GFP-tagged EPN2 (GFP-EPN2) or GFP as a control. We ensured that GFP-EPN2 localized as fine puncta throughout the cell and concentrated in the Golgi region, as it had been shown previously that this distribution of GFP-EPN2 correlates with functionality (19). We infected both cell lines with WSN-Ren, a recombinant IAV that encodes *Renilla* luciferase instead of HA on segment 4 and, thus, allows quantification of viral infection efficiency by measuring *Renilla* luciferase activity (20). In contrast to the Metascape prediction, we did not observe a restrictive effect of EPN2 on IAV infection; virus growth was identical in both cell lines (Fig. 3H).

In summary, EPN2 and EPN1 levels are reduced by CTSW expression but they do not mediate the proviral function of CTSW. Either one of the other targets plays a major role or a combination of several targets is linked to the role of CTSW in IAV entry. Thus, further studies are required, for which our integration of the TAILS data with data from RNAi screens reveals a list of candidate genes to investigate.

### mCTSW plays a proviral role *in vivo*.

**FIG 4:**
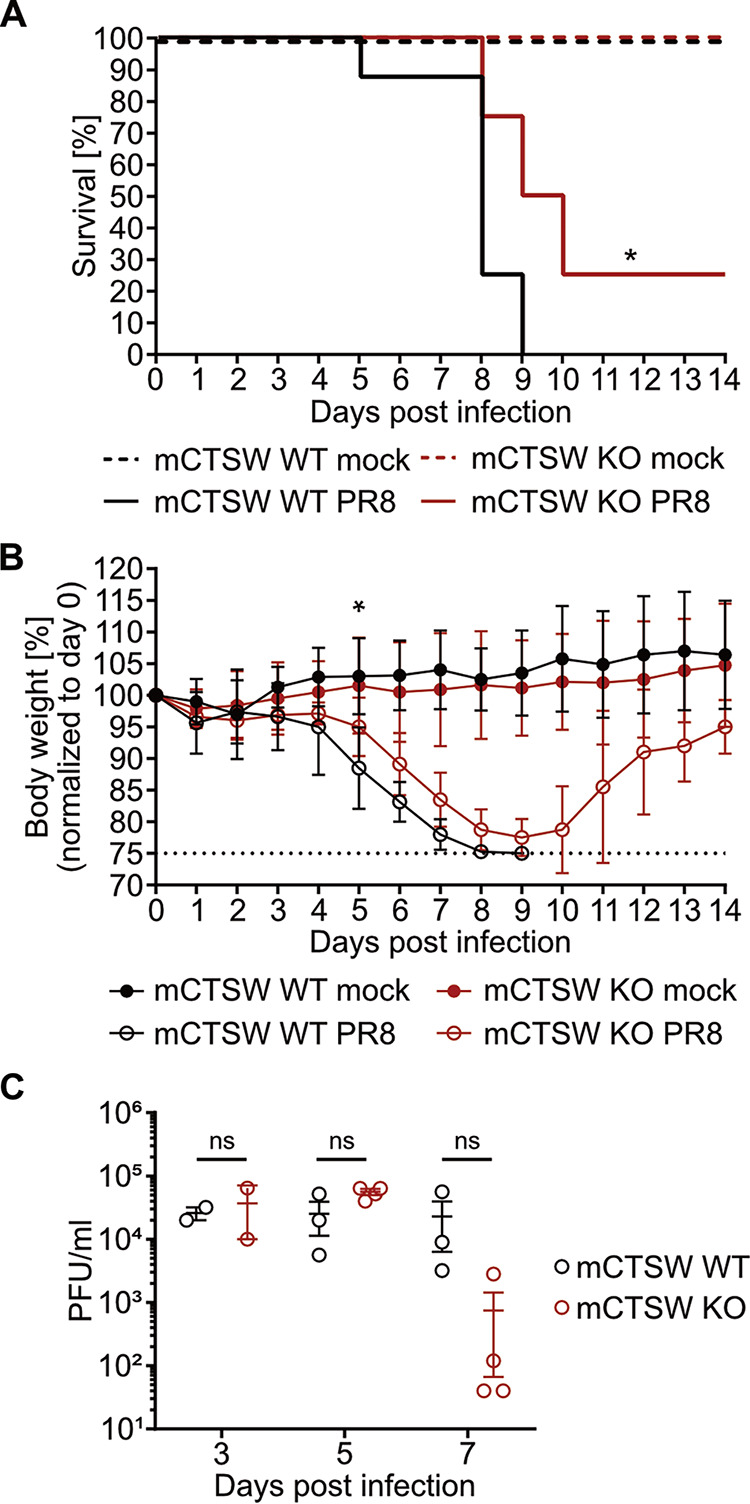
Murine CTSW (mCTSW) plays a proviral role *in vivo*. mCTSW knockout (KO) or wild-type (WT) C57BL/6 mice were intranasally infected with 20 PFU, equivalent to 5 times the median lethal dose (LD_50_), of recombinant influenza virus A/Puerto Rico/8/1934 (PR8) strain or mock infected (*n* = 8 mice/group). (A) Kaplan-Meier survival analysis of infected or mock-challenged mCTSW KO and WT mice. Survival was monitored daily for 14 days, and differences in weight loss were analyzed statistically with the log rank test. *, *P* < 0.05. (B) Percent weight loss from baseline of infected or mock-challenged mCTSW KO and WT mice. Body weight was monitored daily for 14 days. Animals that reached 25% body weight loss met our humane endpoint and were euthanized. For each day, differences between groups were analyzed statistically by 2-way analysis of variance (ANOVA). At a *P* value of <0.05, differences were not significant except for day 5. (C) Viral titers in lung homogenates were determined by plaque assay on days 3, 5, and 7 postinfection. Error bars indicate standard deviations. Significance was assessed with the unpaired *t* test: ns, nonsignificant.

To elucidate the role of CTSW in IAV replication *in vivo*, we proceeded to infect mice deficient in murine CTSW (mCTSW) that had been generated previously and found to be healthy and fertile (21). As mCTSW was found to be highly expressed in CD8^+^ T cells and NK cells, it was speculated that CTSW could be involved in cytotoxic effector cell function. However, this could not be confirmed in subsequent studies and no phenotype has been described so far for the mCTSW knockout (mCTSW KO) mice (21). mCTSW KO or wild-type (mCTSW WT) mice were mock challenged or infected with recombinant IAV (A/Puerto Rico/8/1934), and strikingly, mCTSW KO mice displayed reduced susceptibility to IAV-induced pathogenicity. While all WT mice succumbed to the infection, 25% of mCTSW KO mice survived (Fig. 4A). This statistically significant difference in mortality was accompanied by a delay and reduction in weight loss, as indicated by a statistical difference on day 5 postinfection (Fig. 4B). In line with the observed effects on mortality and weight loss, viral titers in lungs from mCTSW KO mice collected at 7 days postinfection were reduced compared to the viral titers in lungs from mCTSW WT mice (Fig. 4C). However, this trend did not reach statistical significance. Overall, these data suggest a proviral role of mCTSW in IAV infection and, thus, describe the first potential phenotype for the mCTSW KO mice.

## DISCUSSION

CTSW, one of the few cysteine proteases whose function is still unknown, was previously found to play a role in IAV entry, specifically during the fusion step (5). However, the proteolytic targets remained unknown. Herein, we identified potential target proteins of CTSW by testing for proteolytic cleavage of viral proteins and by applying TAILS to reveal cellular targets. Although some cathepsins have been reported to play a role in glycoprotein-dependent entry of different viruses (22–25), the data from this and our earlier study suggest that neither HA nor other IAV proteins are cleaved by CTSW (5). In contrast, the TAILS approach revealed the first set of proteolytic targets of CTSW; in particular, 79 potential direct targets, which were covered by 92 unique N-terminal peptides. Integration with data from RNAi screens for host factors that affect IAV infection efficiency allowed us to highlight potential targets with a predicted impact on IAV infection. From this analysis, epsin 2 (EPN2) stood out, as members of the epsin family play an important role as accessory proteins in clathrin-mediated endocytosis (26, 27), which is the major IAV entry route (17, 18, 28, 29). While TAILS only identified EPN2 as a target, alignment of the peptide sequences around the predicted cleavage site revealed that EPN1, which had been shown to be required for clathrin-mediated endocytosis of IAV (17), could also be a potential CTSW target, and this could be confirmed experimentally. We observed reduced total EPN2 and EPN1 protein levels when coexpressed with CTSW, and this depended on endo-/lysosomal acidification, suggesting CTSW-mediated lysosomal degradation of EPN2 and EPN1. Contrary to our expectations, cotransfection of the enzymatically inactive mutant of CTSW also led to reduced protein levels of EPN2 and EPN1. It might be that endogenous CTSW has a greater effect than expected, and this should be addressed in future studies. Alternatively, it could be speculated that cleavage of EPN2 as measured by TAILS cannot be detected by Western blot analysis, and thus, a TAILS approach with cells expressing catalytically inactive CTSW would be required. However, as we could not confirm the Metascape-predicted restrictive effect of EPN2 or the proviral function of EPN1, it is highly unlikely that the proviral role of CTSW relies heavily on either EPN2 or EPN1, and thus, additional targets and possibly combinations of targets will have to be tested for their role in IAV entry. Moreover, CTSW target proteins not identified in RNAi screens for host factors of IAV should also be considered for future studies.

In addition to the identification of potential CTSW substrates, we also determined the cleavage site specificity of CTSW, which is of crucial importance to elucidate its proteolytic mechanism. CTSW harbors prime site and non-prime site specificities, with most amino acids tolerated in the P2 position. Intriguingly, CTSW clearly favors arginine (N) at the P1′ and lysine (K) at the P1 position when only including the 19 top-ranked peptides identified by TAILS. The specificity information for CTSW can be used to design highly sensitive and specific fluorogenic substrates to establish an *in vitro* protease activity assay. Moreover, the CTSW cleavage site motif can be applied to generate substrate analogs that can act as potent small-molecule inhibitors (30). Currently, four FDA-approved anti-influenza drugs are recommended by the Centers for Disease Control and Prevention (CDC) for treatment of influenza: peramivir, zanamivir, oseltamivir, and the most recent, baloxavir marboxil (31). The M2 inhibitors amantadine and rimantadine have been approved for the treatment and prevention of IAV infection, but their use is no longer recommended due to widespread resistance of many influenza virus strains (32, 33). The rapid emergence of resistance to current anti-influenza drugs has raised a pressing need for the development of new influenza antivirals that can work alone or synergistically with current therapeutics. Targeting host factors instead of viral proteins is expected to diminish or slow down the emergence of resistance (34). Moreover, this option increases the number of potential drug targets substantially, compared to viral targets only. The drawback of this strategy is that targeting host proteins can come with toxicity issues, and this needs to be investigated thoroughly. Our *in vitro* data demonstrate that the host cell protease CTSW is required for IAV replication but not for host cell viability. In line with this, mCTSW KO mice are viable and healthy, which is fundamental for a host factor functioning as a drug target. Strikingly, our *in vivo* data suggested a proviral role of CTSW in IAV infection, rendering CTSW a suitable target for host-directed antivirals. However, the reduced morbidity and mortality were not linked to significant changes in viral titers at early time points of infection, and thus, the proviral mechanism of CTSW *in vivo* needs to be further elucidated. Overall, our work will support the development of novel antivirals to fight influenza.

## MATERIALS AND METHODS

### Chemicals and reagents.

The following compounds were used: dimethyl sulfoxide (DMSO) (Sigma-Aldrich) and bafilomycin A1 (Sigma-Aldrich). LysoTracker red DND-99 (Thermo Fisher Scientific) was used to label acidic organelles in live cells. Peptide-*N*-glycosidase F (PNGase F) (New England Biolabs) was used to remove N-linked oligosaccharides from proteins. For Western blotting and immunofluorescence, antibodies to the following proteins were used as primary antibodies: GAPDH (glyceraldehyde-3-phosphate dehydrogenase) (sc-25778; Santa Cruz Biotechnology), β-actin (sc-47778; Santa Cruz Biotechnology), HA tag (3724; Cell Signaling Technology), V5 tag (MCA1360; Bio-Rad), FLAG M2 tag (F1804; Sigma-Aldrich), 6×His tag (ab18184; Abcam), GFP (ab290; Abcam), epsin 2 (sc-376788; Santa Cruz Biotechnology), epsin 1 (sc-55556; Santa Cruz Biotechnology), CTSW (WH0001521M1; Sigma-Aldrich), calnexin (ab22595; Abcam), histone H3 (ab1791; Abcam), HA (35), NP (ATCC HB-65, H16-L10-4R5; American Type Culture Collection), and NA (PA532238; Thermo Fisher Scientific).

### Plasmids.

The plasmids encoding HA and NA of strain A/WSN/33 in the backbone of the pCAGGS vector have been described before (36, 37). To express wild-type or catalytically inactive CTSW (CTSW WT and CTSW mut, respectively), the constructs pLVX-CTSW(WT, siRes#2)-IRES puro and pLVX-CTSW(C153A, siRes#2)-IRES puro were used (5). For purification of CTSW from cell extracts and supernatants, pcDNA3.1-CTSW-V5-His was cloned by using primers 5′ GATCGGTACCACCATGGCACTGACTGCCCACC 3′ and 5′ GATCCTCGAGGGGAGGGCAGGAGACTCGG 3′ with pCAGGS-CTSW (5) as the template and pcDNA3.1/V5-His A (Thermo Fisher) as the vector. To purify CTSW mut from cell extracts and supernatants, pcDNA3.1-CTSW(C153A)-V5-His was cloned by GeneArt site-directed mutagenesis (Invitrogen) from template pcDNA3.1-CTSW-V5-His according to the manufacturer’s instructions. All plasmids were sequence verified.

The following plasmids were used to validate TAILS hits: pTRACER LA, a gift from Raymond Birge (Addgene plasmid no. 15003), and pcDNA3-HA-Epsin 2 (Addgene plasmid no. 22241) (19), pEGFPC1-EPSIN 2 (Addgene plasmid no. 22242) (19), and pEGFPC1-EPSIN 1 (Addgene plasmid no. 22228) (38), gifts from Pietro De Camilli. pFRT/TO/HIS/FLAG/HA-HNRNPD was a gift from Markus Landthaler (Addgene plasmid no. 38066) (39), and pcDNA3.1 3xFLAG TPP1 88-544 was a gift from Kathleen Collins (Addgene plasmid no. 53548) (40). To assess the role of EPN2 in IAV infection, pLVX-v5-GFP and pLVX-GFP-EPN2 were used. The former was a gift from Samira Schiefer. The latter was cloned by inserting GFP-EPN2 (from pEGFPC1-EPSIN 2) into pLVX-IRES-puro using NheI or SpeI, respectively, and BamHI restriction sites. The constructs were sequence verified.

### Cell lines.

Human embryonic kidney (HEK293T) (ATCC) and human lung adenocarcinoma (A549) (ATCC) cells were cultured in Dulbecco’s modified Eagle’s medium (DMEM) (Thermo Fisher Scientific) supplemented with 10% (vol/vol) fetal bovine serum (FBS) (Thermo Fisher Scientific), 100 units/mL penicillin, and 100 μg/mL streptomycin (Thermo Fisher Scientific) at 37°C with 5% CO_2_ and controlled humidity.

To generate cell lines stably expressing the gene of interest, lentiviral particles were produced by cotransfecting HEK293T cells with the respective pLVX-IRES-puro construct (Clontech), pCMVdR8.91, and pMD2.G (Addgene plasmid no. 12259, a kind gift from Didier Trono) using FuGENE HD transfection reagent (Promega) at a 3:1 transfection reagent/DNA ratio. Supernatants containing lentiviral particles were collected at 48 and 72 h posttransfection, and pooled supernatants were centrifuged at 300 × *g* for 5 min, filtered through a 0.45-μm filter, and used to transduce A549 cells in the presence of 8 μg/mL Polybrene (Sigma-Aldrich). At 48 to 72 h after transduction, cells were subjected to puromycin (InvivoGen) selection (1 μg/mL), which was maintained during subsequent culture.

### Viruses and infection.

IAV strain A/WSN/33 (WSN) was grown on Madin-Darby canine kidney (MDCK) cells, and IAV reporter virus WSN-Ren was propagated on MDCK-HA cells overexpressing the HA protein of strain A/WSN/33 (20, 36). Cells were infected at the indicated multiplicity of infection (MOI) in PBS for infection (PBSi) (Dulbecco’s phosphate-buffered saline [DPBS; Thermo Fisher Scientific] supplemented with 0.3% bovine serum albumin [BSA] [Sigma-Aldrich], 1 mM Ca^2+^ Mg^2+^, 100 units/mL penicillin, and 100 μg/mL streptomycin [Thermo Fisher Scientific]) for 1 h at 37°C or for 1 h on ice for synchronized infection. After removing the inoculum and washing the cells with DPBS, they were overlaid with postinfectious DMEM (piDMEM) (DMEM [Thermo Fisher Scientific] supplemented with 0.1% FBS [Thermo Fisher Scientific], 0.3% BSA [Sigma-Aldrich], 20 mM HEPES [Thermo Fisher Scientific], 100 units/mL penicillin, and 100 μg/mL streptomycin [Thermo Fisher Scientific] containing 1 μg/mL TPCK [tosylsulfonyl phenylalanyl chloromethyl ketone]-treated trypsin [Sigma-Aldrich]) for the indicated times. For WSN-Ren infections, piDMEM was supplemented with 6 μM *Renilla* luciferase substrate (EnduRen live-cell substrate; Promega) and real-time luminescence measurements were taken at the indicated time points using an EnVision multilabel reader (Perkin Elmer) or a Dynex MLx luminometer (Dynex Technologies).

### Transient plasmid transfection.

HEK293T or A549 cells were (co)transfected with 150 ng to 1 μg of each indicated construct using FuGENE HD transfection reagent (Promega) at a 3:1 transfection reagent/DNA ratio. At 24 h posttransfection, cells were treated as indicated.

### siRNA transfection.

A549 cells (30,000 cells per 24-well plate) were reverse transfected with 30 nM siRNA (Qiagen) diluted in Opti-MEM (Thermo Fisher Scientific) using Lipofectamine RNAiMax (Thermo Fisher Scientific) according to the manufacturer’s instructions. After 48 h, the transfection efficiency was verified by cell death of cells treated with an siRNA targeting RPS27A. The following siRNAs were used: scrambled1777 (custom siRNA), 5′-AAGCGTTCGTCCTATGATCGA-3′; siCtsW #2 (SI00025494), 5′-CACCGTGACCATCAACATGAA-3′; siCtsW #3 (SI00025501), 5′-CGCGTTCATAACTGTCCTCAA-3′; siRPS27A (custom siRNA), 5′-AAGCTGGAAGATGGACGTACT-3′; siEPN1 #2 (SI00380604), 5′-TCCCGCGACGCTCACCCTGAA-3′; siEPN1 #4 (SI00380618), 5′-CCGGAAGACGCCGGAGTCATT-3′; and siEPN1 #6 (SI04996789), 5′-CGGTGAATCCTTGGTGATGAT-3′.

### CTSW purification from cell extracts and supernatants.

Four 6-well plates of HEK293T cells were transfected with pcDNA3.1-CTSW-V5-His or pcDNA3.1-CTSW(C153A)-V5-His for 72 h. Supernatants were harvested and precleared by centrifugation at 4,700 rpm for 10 min. Cells were washed with PBS, and 300 μL NP-40 (1%) was added to each well to lyse the cells. Lysates were pooled and filled up to 40 mL with NP-40 (1%). Supernatants and cell extracts were cleared by filtration (0.22-μm pore size). Nickel Sepharose 6 fast-flow resin was prepared according to the manufacturer’s instructions and incubated with cell extracts or supernatants for 5 h at 18°C with shaking at 100 rpm. Cleared supernatants/cell extracts were discarded, and resin was loaded onto a column. After five washes with Ni wash buffer 2 (50 mM NaH_2_PO_4_, 300 mM NaCl, 40 mM imidazole, pH 8), 10 mL of Ni elution buffer (50 mM NaH_2_PO_4_, 300 mM NaCl , 250 mM imidazole, pH 8) was added to elute the purified protein. Protein was concentrated using a 10-kDa Amicon tube (Merck) and centrifugation at 4,000 rpm for 30 min at 18°C.

### TAILS.

After siRNA-mediated knockdown (KD) of CTSW in A549 cells, CTSW wild-type (WT) and CTSW KD cells were infected with A/WSN/1933 (MOI of 10 PFU/mL) for 1 h at 4°C, followed by 90 min of incubation at 37°C. After subcellular fractionation using the Qproteome cell compartment kit (Qiagen), the membrane fractions of 10 samples (5 replicates of CTSW WT and 5 replicates of CTSW KD) were processed at the Functional Genomics Center Zurich (FGCZ), following the terminal amine isotopic labeling of substrates (TAILS) protocol (11). Briefly, samples were precipitated by acetone/methanol precipitation (100 μg per sample), reduced, and alkylated and all natural and cleaved N-terminal and lysine residues were blocked by tandem mass tag (TMT) labeling (TMT10plex isobaric mass tagging kit; Thermo Fisher Scientific). Pooled samples were then digested with trypsin for 6 h at 42°C. After digestion, aliquots were taken and designated as pre-TAILS samples (after digestion, before pull-out). In order to reduce sample complexity, an aldehyde-derivatized amine-reactive polymer (HPG-ALDII) was added to remove all unlabeled peptides. After overnight incubation at 37°C and quenching, the labeled peptides were recovered by spinning through a Microcon-30kDa centrifugal filter unit (Merck Millipore) and designated as TAILS samples (after pull-out). Both the pre-TAILS and the TAILS samples were desalted by C_18_ and injected into an LC-MS/MS system. The protein identification and quantification were performed using Proteome Discoverer 2.3 software against the Homo sapiens and IAV (A/WSN/1933) protein database. The following filters were applied for peptide groups: *P* value of <0.01, log_2_ fold change for KD/WT of ≤−1 or ≥1, modifications contain TMT6plex [N-term], sequence ends with arginine (R), annotated sequence does not start with [-] or [M]. Sequences of identified N-terminal peptides were analyzed by using WebPics (http://clipserve.clip.ubc.ca/pics/) with the following settings: no enzyme used to generate peptide library (TAILS data), human cell culture used to generate peptide library, no subsite cooperativity analysis (41). Proteomic identification of protease cleavage sites (PICS) sequences were formatted for use with the sequence logo software iceLogo (42).

### Western blot analysis.

Cells were lysed in 1× Laemmli buffer (62.5 mM Tris-HCl [pH 6.8], 10% glycerol, 2% SDS, 100 mM dithiothreitol [DTT], and 0.02% bromophenol blue), and cell lysates were homogenized with QIAshredder columns (Qiagen). Samples were boiled at 95°C for 5 min before they were loaded on Bolt 4 to 12% Bis-Tris plus gels (Thermo Fisher Scientific) to perform protein separation by gel electrophoresis. After 20 to 35 min at 200 V, proteins were transferred to nitrocellulose membranes (GE Healthcare Life Sciences) for 70 min at 10 V. Next, membranes were blocked in 5% milk in Tris-buffered saline (20 mM Tris-HCl [pH 7.6] and 150 mM NaCl) with 0.1% Tween 20 (Sigma-Aldrich) (TBS-T) for 1 h at room temperature (RT) before membranes were incubated with appropriate dilutions of primary antibodies in TBS-T either for 2 h at RT or overnight at 4°C. After incubation with conjugated secondary antibodies in TBS-T, signals were developed using an LAS-4000 mini (Fujifilm Life Sciences) or an Odyssey Fc imaging system (LI-COR Biosciences). Signal intensities were quantified using the Image Studio Light software (LI-COR Biosciences).

### Immunofluorescence staining and microscopy.

For confocal microscopy, cells were seeded on glass coverslips in 24-well plates. The next day, cells were fixed with 3.7% paraformaldehyde solution (Electron Microscopy Science) in DPBS for 10 min at RT and permeabilized with 0.5% Triton X-100 (Sigma-Aldrich) in DPBS for 5 min at RT before blocking with 2% FBS (Thermo Fisher Scientific) in DPBS for 30 min at RT. Cells were then incubated with the corresponding primary antibodies diluted in 2% FBS in DPBS for 2 h at RT. After washing three times with DPBS, cells were incubated with Alexa Fluor-conjugated secondary antibodies (Thermo Fisher Scientific) diluted in 2% FBS in DPBS for 1 h at RT on a plate shaker protected from light. Cell nuclei were counterstained with DAPI (4′,6-diamidino-2-phenylindole; Sigma-Aldrich). For confocal microscopy, stained cells were inversely mounted onto glass microscope slides using ProLong gold antifade mountant (Thermo Fisher Scientific). Images were acquired using an SP5 or SP8 confocal laser scanning microscope (Leica Microsystems) or a DM IL LED inverted microscope (Leica Microsystems). The quantification of the nuclear NP signal was performed using ImageJ software.

### Reverse transcription-qPCR analysis.

Cellular RNA was isolated using the ReliaPrep RNA miniprep kit (Promega) according to the manufacturer’s recommendations. Extracted RNA was measured using the Nanodrop spectrometer, and at least 750 ng of RNA was reverse transcribed into cDNA using SuperScript III reverse transcriptase (Thermo Fisher Scientific) and oligo(dT) primers (Promega). Reverse transcription-qPCR was performed using 1:5-diluted cDNA, specific primers (EPN1 forward, 5′-CCCTTAGTTTGAGCCGAGAAGAG-3′, and EPN1 reverse, 5′-AGGTCCATGAGGGACGACTC-3′), and EvaGreen qPCR master mix (Biotium) or PowerTrack SYBR green master mix (Thermo Fisher Scientific) in the 7300 real-time PCR system (Applied Biosystems). Relative gene expression was determined using the cycle threshold (ΔΔ*C_T_*) method with GAPDH serving for normalization.

### Mouse experiments.

mCTSW transgenic mice have been described previously (21). Wild-type C57BL/6 mice were purchased from The Jackson Laboratory (Bar Harbor, ME). Six- to 8-week-old female mice were randomly assigned to experimental groups and housed under specific-pathogen-free (SPF) conditions. All animal studies were reviewed and approved by the Institutional Animal Care and Use Committee (IACUC) of the Icahn School of Medicine at Mount Sinai in accordance with the institutional and national guidelines and regulations and were performed in Association for the Assessment and Accreditation of Laboratory Animal Care (AAALAC)-certified facilities.

Animals were anesthetized using a cocktail of ketamine/xylazine (100/10 mg/kg of body weight) in sterile saline via the intraperitoneal route. Anesthetized animals were then challenged with 20 PFU, equivalent to 5 times the median lethal dose (LD_50_), of the recombinant influenza virus A/Puerto Rico/8/1934 (PR8) in 25 μL of sterile saline via the intranasal route. Control groups were mock challenged with 25 μL of sterile saline via the intranasal route. Mice were monitored daily for body weight, as well as signs of morbidity, for 14 days postinfection. Animals reaching 25% body weight loss met our humane endpoint, and thus, they were euthanized and removed from the experimental cohort. At days 3, 5, and 7 postinfection, selected animals were euthanized and lung tissue samples were collected to assess viral replication. Lung tissue homogenates were obtained by two rounds of mechanical shearing for 10 s at 6.5 m/s in a 450-μL volume of sterile saline per sample, using sterile zirconium beads in 2.0-mL tubes (Alkali Scientific) in a FastPrep24 homogenizer (MP Biomedicals, CA). Tissue debris was removed by sequential low-speed centrifugations. Viral titers in the supernatant were quantified using standard plaque assay techniques in MDCK cells.
