# Supplementary material for: Proteomic Identification of Potential Target Proteins of Cathepsin W for Its Development as a Drug Target for Influenza
Source: Microbiol Spectr. 2022 Jul 14;10(4):e00921-22. doi: 10.1128/spectrum.00921-22 (PMC9431242; doi:10.1128/spectrum.00921-22)
Supplement: Supplemental file 1 — Supplemental material. Download spectrum.00921-22-s0001.pdf, PDF file, 1.4 MB [file spectrum.00921-22-s0001.pdf]

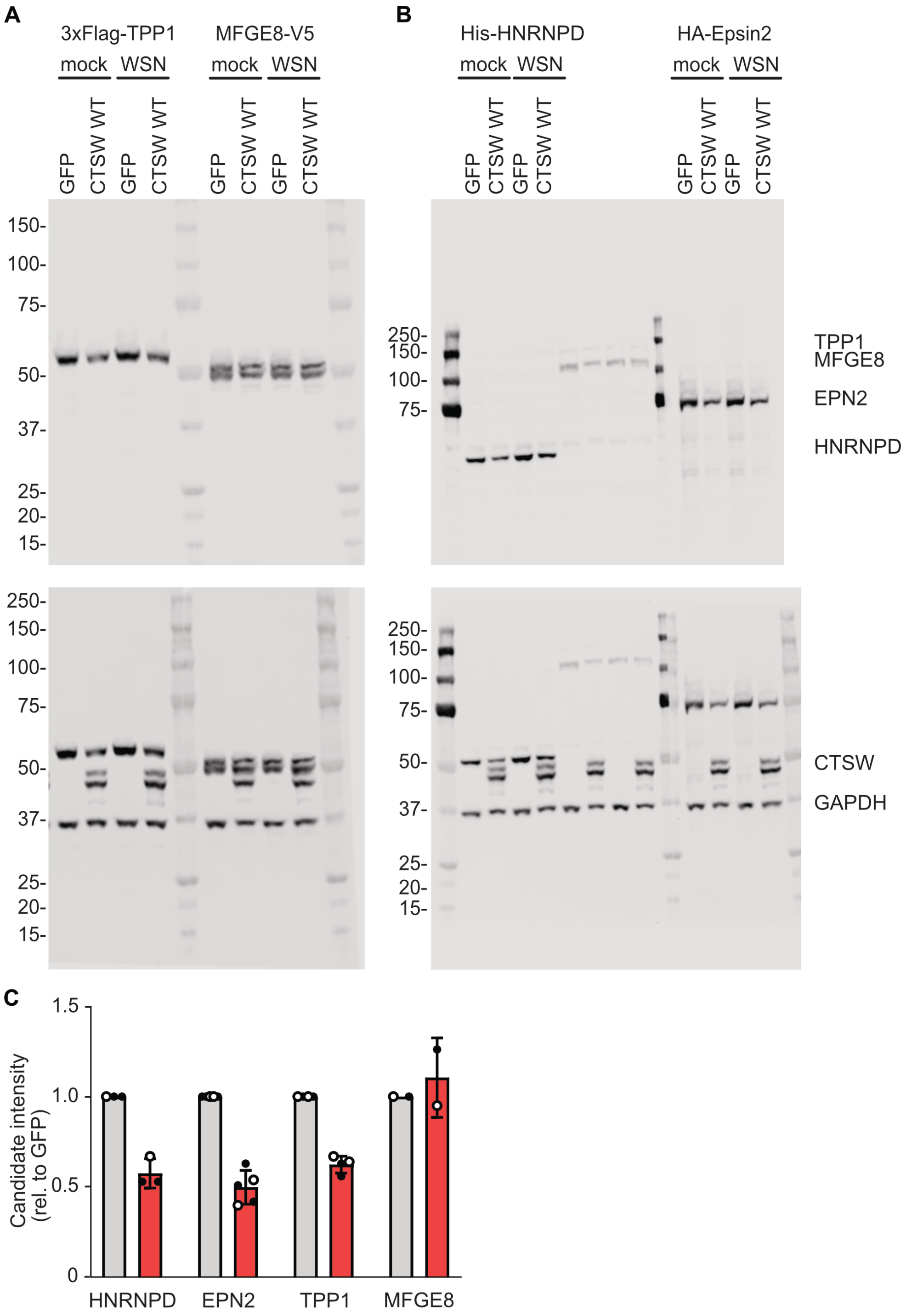
A
3xFlag-TPP1
MFGE8-V5
mock
WSN
mock
WSN
GFP
CTSW WT
GFP
CTSW WT
GFP
CTSW WT
GFP
CTSW WT
150-
100-
75-
50-
37-
25-
20-
15-
250-
150-
100-
75-
50-
37-
25-
20-
15-
B
His-HNRNPD
HA-Epsin2
mock
WSN
mock
WSN
GFP
CTSW WT
GFP
CTSW WT
GFP
CTSW WT
GFP
CTSW WT
250-
150-
100-
75-
TPP1
MFGE8
EPN2
HNRNPD
250-
150-
100-
75-
50-
37-
25-
20-
15-
CTSW
GAPDH
C
Candidate intensity (rel. to GFP)
1.5
1.0
0.5
0
HNRNPD
EPN2
TPP1
MFGE8

1

Supplementary Figure S1. Levels of EPN-2, TPP1, HNRNPD, but not MFGE8 are reduced upon expression of CTSW. (A-B) Original blots from figure 2E are shown. Blots were first stained against the tag of the candidates (top blots), then for CTSW and GAPDH (lower blots). Blots in (B) contain four lanes in the middle that are not relevant to data shown in figure 2E. (C) Band intensities for the target proteins were quantified from multiple independent blots, normalized to GAPDH levels and are given relative to the GFP-expressing control. Grey bars depict relative intensities from GFP-expressing cells (set to 1), red bars show relative intensities for CTSW-expressing cells. Black dots represent results from mock infected cells, white dots from infected cells. Error bars display standard deviation.
